# Complete genomes of 22 bacterial strains isolated from polluted soil microbiota via enrichment on PFAS as a sole carbon source

**DOI:** 10.1128/mra.00182-26

**Published:** 2026-08-20

**Authors:** Gabriele Bellotti, Sonia Gaaied, Filippo Vaccari, Nicoleta Alina Suciu, Giancarlo Renella, Antonio Masi, Edoardo Puglisi

**Affiliations:** 1 Department for Sustainable Food Process (DiSTAS), Università Cattolica del Sacro Cuore 9371, Piacenza, Province of Piacenza, Italy; 2 Department of Agronomy, Food, Natural Resources, Animals and Environment (DAFNAE), University of Padova, Legnaro, Padua, Italy; University of Wisconsin-Madison, Madison, Wisconsin, USA

**Keywords:** soil enrichment culture, heptafluorobutyric acid (HFBA), perfluorooctanoic acid (PFOA), xenobiotic, long-read assembly, environmental isolates, Veneto region, Italy

## Abstract

A total of 22 bacterial strains were isolated from PFAS-contaminated soil (Veneto, Italy), after a 5-month enrichment using perfluorooctanoic acid and heptafluorobutyric acid. Whole genomes were sequenced and screened with a curated database of dehalogenase-related proteins. All genomes showed potential for fluorinated compound transformation.

## ANNOUNCEMENT

Per- and polyfluoroalkyl substances (PFAS) are complex and ubiquitous pollutants (1). Their strong C–F bonds make PFAS highly resistant to biodegradation, with known toxic bioaccumulative properties in higher animals (2–4). As a result, environmental remediation of PFAS-contaminated sites remains a major challenge.

In this announcement, we report the complete genomes of 22 bacteria isolated from soil collected at a heavily PFAS-contaminated site in the Veneto region, Italy (45.551389°N, 11.387500°E). The area is affected by legacy pollution, with reported total PFAS concentrations exceeding 1,000 ng/kg in soil and 2,000 ng/L in water (5). Soils were collected from the top 0 to 10 cm layer using a stainless-steel auger and transported on ice to the laboratory.

Enrichment cultures were prepared by inoculating 1 g of soil into 100 mL of M9 mineral medium with perfluorooctanoic acid (PFOA) or heptafluorobutyric acid (HFBA) (50 mg/L each) as the sole carbon source and incubated at 30°C with monthly transfers into fresh medium over 5 months.

After the final transfer, bacteria were isolated on R2A agar, purified by repeated streaking, and confirmed as pure and unique isolates via rep-PCR using (GTG)_5_ and M13 primers (6, 7), yielding 22 isolates able to grow with PFOA or HFBA. Pure strains were stored in 20% glycerol at −45°C for further analysis.

For whole-genome sequencing, isolates were grown in LB for 48 h, and genomic DNA was extracted using the E.Z.N.A. Bacterial DNA Kit (Omega Bio-tek, USA). DNA integrity and concentration were assessed by agarose gel electrophoresis and Qubit 2.0 fluorometer (Invitrogen, UK), respectively. Libraries were prepared using the Rapid Barcoding Kit 24 v14 (SQK-RBK114.24, ONT, UK) following the manufacturer’s instructions. No additional DNA shearing or size-selection step was performed prior to library preparation. Libraries were loaded on an ONT MinION Mk1B device and sequenced using a FLO-MIN114 (R10.4.1) flow cell with MinKNOW software v23.11.5 (https://nanoporetech.com/software/devices/minion-mk1b/software). Basecalling was performed with Dorado v0.5.3 (https://github.com/nanoporetech/dorado). Generated high-fidelity FASTQ reads were filtered with Nanofilt v2.8.0 (8), retaining sequences >1,000 bp with a Phred score >10.

Default parameters were used for all software unless otherwise noted. Genome assemblies were generated using three long-read assemblers, Flye v2.9.6 (9), Raven v1.8.3 (10), and Miniasm v0.3 r179 (11). Trycycler v0.5.5 (12) was then used to reconcile assemblies, resolve structural discrepancies, and circularize replicons by detecting and trimming end-to-end overlapping sequences and rotating to a consistent start position, finally generating the consensus assembly, including putative plasmids when present. Complete genome assemblies were annotated using Prokka v1.2.0 (13). Descriptive metrics for raw reads and genomes are summarized in Table 1.

**TABLE 1:** Genome assembly summary data

Strain ID	Biosample	Total number of reads	Read length range (min–max bp)	Total sequence data (nt)	Total number of contigs	Genome size (Mb)	N50 (bp)	Assembly GC content (%)	Nanopore coverage	Predicted genes (total)	NCBI taxonomy	CheckM completeness (%)	CheckM contamination (%)	Assembly accession	SRA raw data accession
UC4457	SAMN50934426	138.4k	1,000–92,296	456.7M	1	4.3	4,307,368	67	101×	3,964	*Pseudoxanthomonas beigongshangi*	93.02	6.99	GCA_052978125.1	SRR37104826
UC4454	SAMN50934424	87.2k	1,000–82,459	479.8M	3	5.1	3,491,798	64.5	94×	4,968	*Aquamicrobium terrae*	98.22	1.16	GCA_052978165.1	SRR37104825
UC4435	SAMN50934407	137.5k	1,000–107,766	942.3M	1	2.5	2,547,873	73	338×	2,380	*Micrococcus luteus*	98.7	0.46	GCA_052978465.1	SRR37104814
UC4434	SAMN50934406	82.8k	1,000–71,334	356.3M	1	2.5	2,524,094	73	136×	2,339	*Micrococcus luteus*	98.7	0.46	GCA_052978485.1	SRR37104811
UC4458	SAMN50934427	105.6k	1,000–105,721	577.0M	1	7	6,975,423	66	83×	6,444	*Achromobacter anxifer*	99.05	1.37	GCA_052978085.1	SRR37104810
UC4456	SAMN50934425	101.6k	1,000–115,683	581.8M	1	6.9	6,923,855	66.5	83×	6,399	*Achromobacter anxifer*	98.82	1.14	GCA_052978145.1	SRR37104809
UC4444	SAMN50934415	124.6k	1,000–72,358	444.7M	1	3.9	3,884,253	67.5	116×	3,538	*Rhodanobacter* sp.	84.5	6.89	GCA_052978305.1	SRR37104808
UC4437	SAMN50934409	91.5k	1,000–79,195	527.1M	1	3.9	3,884,253	67.6	138×	3,537	*Rhodanobacter* sp.	84.5	6.89	GCA_052978425.1	SRR37104807
UC4443	SAMN50934414	136.2k	1,000–107,214	744.7M	1	3.8	3,829,296	67.5	198×	3,457	*Rhodanobacter* sp.	84.75	5.35	GCA_052978325.1	SRR37104806
UC4436	SAMN50934408	135.1k	1,000–97,627	748.5M	1	3.8	3,829,295	67.5	193×	3,456	*Rhodanobacter* sp.	84.83	5.35	GCA_052978445.1	SRR37104805
UC4439	SAMN50934411	38.2k	1,000–73,996	149.0M	3	3.8	3,826,438	67.5	38×	3,516	*Rhodanobacter* sp.	84.26	7.56	GCA_052978385.1	SRR37104824
UC4448	SAMN50934418	72.4k	1,000–69,164	266.7M	1	3.8	3,826,436	67.5	69×	3,497	*Rhodanobacter* sp.	84.4	7.04	GCA_052978265.1	SRR37104823
UC4438	SAMN50934410	342.6k	1,000–105,960	1.6G	1	3.8	3,826,435	67.5	234×	3,498	*Rhodanobacter* sp.	84.4	7.04	GCA_052978405.1	SRR37104822
UC4452	SAMN50934422	209.9k	1,000–104,437	990.9M	1	3.8	3,826,434	67.5	259×	3,494	*Rhodanobacter* sp.	84.4	7.04	GCA_052978205.1	SRR37104821
UC4450	SAMN50934420	122.4k	1,000-123,000	813.4M	1	3.8	3,826,433	67.5	216×	3,497	*Rhodanobacter* sp.	84.4	6.59	GCA_052978245.1	SRR37104820
UC4451	SAMN50934421	43.4k	1,000–136,569	238.7M	1	3.8	3,788,358	67.5	62×	3,409	*Rhodanobacter* sp.	84.12	6.49	GCA_052978225.1	SRR37104819
UC4442	SAMN50934413	106.4k	1,000–97,875	409.2M	1	3.3	3,255,298	63.5	127×	3,126	*Castellaniella* sp.	98.17	0.71	GCA_052978345.1	SRR37104818
UC4447	SAMN50934417	80.1k	1,000–92,694	444.3M	1	3.3	3,255,296	63.5	138×	3,127	*Castellaniella* sp.	98.17	0.71	GCA_052978285.1	SRR37104817
UC4453	SAMN50934423	98.1k	1,000–95,315	686.9M	1	3.2	3,182,604	66	216×	3,119	*Allosphingosinicella humi*	99.26	1.31	GCA_052978185.1	SRR37104816
UC4440	SAMN50934412	71.6k	1,000–220,965	333.8M	1	2.4	2,402,549	65	73×	2,316	*Corynebacterium sanguinis*	99.55	0	GCA_052978365.1	SRR37104815
UC4446	SAMN50934416	35.6k	1,000–91,011	199.6M	2	5.3	4,966,310	62.5	37×	5,076	*Nitrobacteraceae* bacterium	below_threshold_lineage_match	below_threshold_lineage_match	GCA_053851815.1	SRR37104813
UC4449	SAMN50934419	42.6k	1,000–108,043	339.3M	2	5.3	4,943,793	62.5	65×	5,025	*Nitrobacteraceae* bacterium	below_threshold_lineage_match	below_threshold_lineage_match	GCA_053851795.1	SRR37104812

A maximum-likelihood phylogenetic tree was inferred from a concatenated alignment of 383 single-copy core genes identified across 22 genomes using RAxML v8.2.12 implemented in BV-BRC phylogenetic tree service (14, 15).

**Fig 1:**
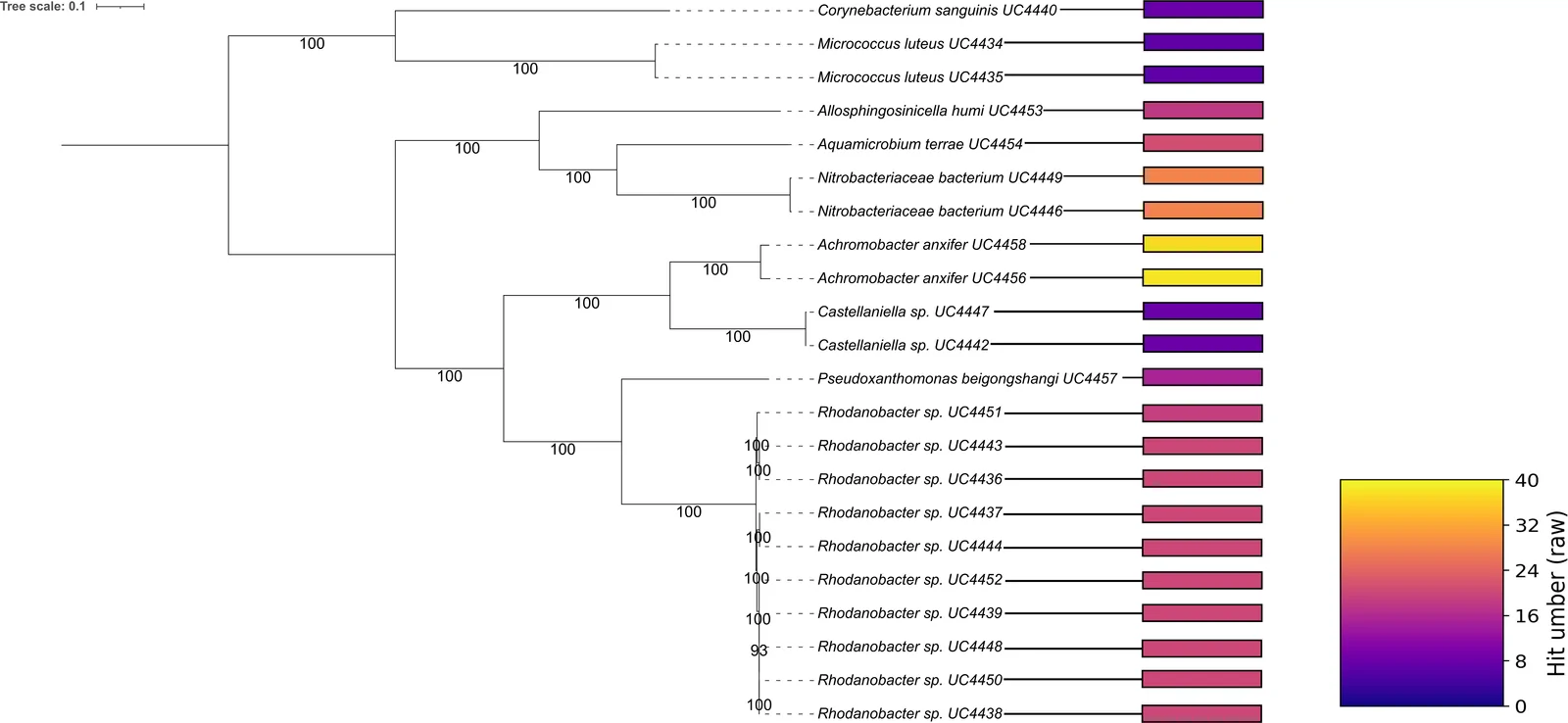
Phylogenetic tree of the isolates obtained in this study inferred with 100 bootstrap using RAxML and visualized using iTol v7 (17). Numbers at nodes indicate bootstrap support (%), and the scale bar represents substitutions per site. The heatmap (right) shows, for each isolate, the raw number of dehalogenase-related gene hits detected in the genome.

A custom database was built with DIAMOND v2.1.8 (16) from 5,248 manually curated UniProt sequences associated with dehalogenation, putative defluorination, and fluoride metabolism. Genomes were screened using BLASTP, retaining hits with *e*-value ≤1 × 10⁻¹⁰, identity ≥35%, coverage ≥100 amino acids. Putative dehalogenation- or defluorination-related genes were detected in all isolates (Fig. 1).

## Data Availability

This Whole-Genome Shotgun project named “Complete genomes of 22 bacterial strains isolated from polluted soil microbiota via enrichment on PFAS as a sole carbon source” has been deposited on NCBI under BioProject accession PRJNA1314445. Raw reads and assemblies are available under the accession numbers reported in Table 1. The curated list of protein sequences used for homology-based screening and for construction of the custom DIAMOND database is available in FASTA format from the Zenodo repository: https://doi.org/10.5281/zenodo.20611709. This file includes the sequence identifiers and amino acid sequences used to screen the genomes for dehalogenation-, putative defluorination-, and fluoride metabolism-related proteins.
